# Bumblebee Workers Show Differences in Allele-Specific DNA Methylation and Allele-Specific Expression

**DOI:** 10.1093/gbe/evaa132

**Published:** 2020-06-29

**Authors:** Hollie Marshall, Alun R C Jones, Zoë N Lonsdale, Eamonn B Mallon

**Affiliations:** Department of Genetics and Genome Biology, University of Leicester, United Kingdom

**Keywords:** bumblebee, Hymenoptera, genomic imprinting

## Abstract

Allele-specific expression is when one allele of a gene shows higher levels of expression compared with the other allele, in a diploid organism. Recent work has identified allele-specific expression in a number of Hymenopteran species. However, the molecular mechanism which drives this allelic expression bias remains unknown. In mammals, DNA methylation is often associated with genes which show allele-specific expression. DNA methylation systems have been described in species of Hymenoptera, providing a candidate mechanism. Using previously generated RNA-Seq and whole-genome bisulfite sequencing from reproductive and sterile bumblebee (*Bombus terrestris*) workers, we have identified genome-wide allele-specific expression and allele-specific DNA methylation. The majority of genes displaying allele-specific expression are common between reproductive and sterile workers and the proportion of allele-specific expression bias generally varies between genetically distinct colonies. We have also identified genome-wide allele-specific DNA methylation patterns in both reproductive and sterile workers, with reproductive workers showing significantly more genes with allele-specific methylation. Finally, there is no significant overlap between genes showing allele-specific expression and allele-specific methylation. These results indicate that *cis*-acting DNA methylation does not directly drive genome-wide allele-specific expression in this species.

## Introduction

Allele-specific expression is when one allele of a gene shows higher levels of expression compared with the other allele in a diploid organism. It has been associated with genomic mechanisms such as X-chromosome inactivation and genomic imprinting, that is, parent-of-origin allele-specific expression (Knight 2004). Epigenetic mechanisms such as DNA methylation and histone modifications have been associated with the production of allele-specific expression, for example, in mammals and angiosperm plants imprinted genes are usually associated with allele-specific DNA methylation (Barlow and Bartolomei 2014). Additionally, epigenetic mechanisms such as histone modifications and DNA methylation are thought to play a role in the inactivation of the entire paternal chromosome in some insect species from the *Sciaridae* and *Coccoideae* families (Prantera and Bongiorni 2012). Recently, parent-of-origin allele-specific expression has been identified in two Hymenoptera species, the honeybee (Kocher et al. 2015; Galbraith et al. 2016; Smith et al. 2020) and the buff-tailed bumblebee (Marshall et al. 2020). However, the mechanism by which genes exhibit general allele-specific expression bias in insects remains unknown.

Many insects have functional DNA methylation systems, including the honeybee (Lyko et al. 2010; Bewick et al. 2016) and bumblebee (Sadd et al. 2015) mentioned above. However, the function of DNA methylation in insects remains debated (Glastad et al. 2019). Various studies have found an association between methylation and gene expression (Bonasio et al. 2012; Glastad et al. 2014; Marshall et al. 2019), and alternative splicing (Lyko et al. 2010; Glastad et al. 2016) in social insects. However, multiple other studies have found no such association (Patalano et al. 2015; Arsenault et al. 2018), with Libbrecht et al. (2016) concluding that many previous studies on caste-specific DNA methylation have been confounded by individual methylation variation. The association between allele-specific expression and methylation is also unclear. Allele-specific expression has been associated with allele-specific methylation in two ant species, *Camponotus floridanus* and *Harpegnathos saltator* (Bonasio et al. 2012). However, another study did not find a genome-wide relationship between allele-specific expression and methylation in a hybrid cross of two nonsocial wasp species, *Nasonia vitripennis* and *Nasonia giraulti* (Wang et al. 2016). These many conflicting studies leave the regulatory capacity of DNA methylation in insects debated, specifically in terms of the role of allele-specific methylation in regulating allele-specific expression.

It is also worth noting there have been a number of nonimprinted loci found to show allele-specific expression in various species, these genes have been directly associated with *cis*-acting polymorphic sites, such a single-nucleotide polymorphisms (SNPs) (Tycko 2010; Wang et al. 2016). This has also been the case for genes showing allele-specific methylation, where the methylation status of an allele can be dependent on the underlying genotype (Kerkel et al. 2008; Remnant et al. 2016; Wang et al. 2016). Additionally, there are a number of genes identified in humans which show apparently random allele-specific expression, that is, some cell types express one allele, whereas others express the other copy and some express both alleles (Gimelbrant et al. 2007).

Bumblebees provide an ideal system to further investigate the relationship between allele-specific methylation and allele-specific expression in insects. Using a candidate gene approach, previous research identified allele-specific expression in a gene (ecdysone 20-monooxygenase-like) related to worker reproductive behavior in *Bombus terrestris* (Amarasinghe et al. 2015). Additional research has since used RNA-seq data to identify >500 loci showing allele-specific expression throughout the *B. terrestris* genome (Lonsdale et al. 2017). This same study also identified 19 genes displaying allele-specific expression and allele-specific methylation, although this was in a single individual (Lonsdale et al. 2017). Although this study laid the groundwork for further investigation, it is still unknown to what extent genome-wide allele-specific methylation is maintained across individuals, colonies, and reproductive worker states.

In order to identify the genome-wide relationship between allele-specific expression and allele-specific methylation in *B. terrestris*, we have taken advantage of a previously generated data set. These data consist of whole-genome bisulfite sequencing and RNA-seq from reproductive and sterile workers, spanning three genetically distinct colonies. We hypothesize that if DNA methylation plays a causative role in the generation of allele-specific expression then we will identify genes which display both allele-specific methylation and expression. If the underlying genotype affects allele-specific expression then we expect to see differences between colonies which are not associated with DNA methylation. Finally, if allele-specific expression and/or allele-specific methylation are relatively stable then we would expect to see few differences between reproductive and sterile workers.

## Materials and Methods

### Samples and Data

The data used in this study were generated in previously published work by Marshall et al. (2019). Briefly, these consist of 18 RNA-Seq libraries generated from head tissue of three reproductive workers and three sterile workers per colony, with three independent colonies total. DNA from head tissue from the same individuals was pooled by reproductive status and colony for whole-genome bisulfite sequencing, producing one representative reproductive sample and one sterile sample per colony replicate, giving six whole-genome bisulfite libraries total. One RNA-Seq sample, J8_24, was excluded from this study as it was possibly incorrectly labeled in the previous work (see Marshall et al. [2019]).

### Identification of Allele-Specific Expression

RNA-Seq data were quality checked using fastqc v.0.11.5 (Andrews 2010) and trimmed using CutAdapt v1.1 (Martin 2011). Trimmed data were aligned to the reference genome (Bter_1.0, Refseq accession no. GCF_000214255.1; Sadd et al. 2015) using STAR v2.5.2 (Dobin et al. 2015) with standard parameters. SNPs were then called from the RNA-Seq library of each sample following the GATK best practices for SNP calling from RNA-Seq data (Auwera 2014). Briefly, this involves assigning read groups and marking duplicate reads using Picard v.2.6.0 (Broad Institute 2018), removing reads overlapping introns to keep only exonic reads, calling SNPs with a minimum confidence score of 20.0, then filtering SNPs by windows of three within a 35-bp region, to keep only those with a Fisher strand value >30.0 and a quality by depth value >2.0 (these filtering steps are considered particularly stringent) (Auwera 2014). These SNPs were then incorporated into the WASP v.0.3.1 pipeline (van de Geijn et al. 2015) which remaps all reads with either the reference SNP or alternative SNP in order to reduce reference allele mapping bias. Reads that cannot be mapped with the alternative SNP are discarded. SNPs were then filtered to keep only biallelic SNPs allowing individual alleles to be identified. Final reads were then counted per biallelic SNP using the “ASEreadcounter” program from GATK.

**Fig. 1.**
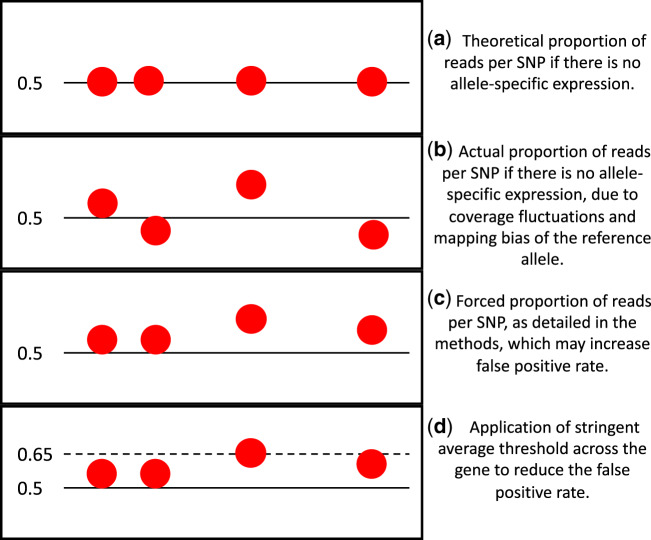
Overview of the theoretical proportions of reads per SNP in a gene which does not show allele-specific expression. Each red dot is an individual SNP.

A custom R script was used to annotate the SNP positions with gene identifiers, SNPs were filtered to remove those with a coverage of <10. SNPs were also removed if they had a count of zero for either the alternative or reference SNP as they may have been mis-called by the SNP caller as heterozygous when they are actually homozygous. Two new columns were then created to represent each allele, as we do not have parental genomes it is not possible to tell which SNPs belong to which allele (e.g., a reference SNP at a given position may be accompanied with an alternative SNP on the same allele). The counts for each SNP were then allocated to either “allele: 1” or “allele: 2,” with the highest counts per SNP allocated to “allele: 1” (fig. 1 and supplementary 2.0, fig. S1, Supplementary Material online). Counts per SNP per allele were then summed over each gene for each reproductive status per colony creating one representative sample per reproductive status per colony. Conducting analyses on a per gene basis decreases false-positive calls of allele-specific expression which may occur if there is some remaining reference allele mapping bias after remapping with WASP (Degner et al. 2009). This method is necessary as we are not looking for parent-of-origin expression and so do not have the parental genomes available to determine maternal/paternal alleles.

As this method is naive to allele-specific alternative splicing, stringent filtering was applied throughout. Only genes with counts found in at least two of the three colony replicates per reproductive or sterile workers were tested. A logistic regression model was then applied with the proportion of allelic expression per gene as the dependent variable and with reproductive status and colony as independent variables, a quasibiomial distribution was applied to account for any overdispersion within the data. *P* values were corrected for multiple testing using the Benjimini–Hochberg method (Benjamini and Hochberg 1995) and genes were classed as showing allele-specific expression if the *q* value was <0.05 and the average proportion of allelic expression per reproductive state across colonies was >0.65. This stringent filtering was used to account for cases of mis-allocation of SNPs to the correct alleles (fig. 1).

### Identification of Allele-Specific Methylation

Whole-genome bisulfite sequencing data quality was checked using fastqc v.0.11.5 (Andrews 2010) and trimmed using CutAdapt v1.1 (Martin 2011). Trimmed data were aligned to the reference genome (Bter_1.0, Refseq accession no. GCF_000214255.1; Sadd et al. 2015) using Bismark v.0.16.1 (Krueger and Andrews 2011) and bowtie2 v.2.2.6 (Langmead and Salzberg 2012) with standard parameters. Alignment output files were deduplicated using Bismark v.0.16.1 (Krueger and Andrews 2011) and sorted and indexed using samtools v.1.3.2 (Li et al. 2009).

Allele-specific methylation was determined using a probabilistic model implemented using the “*amrfinder*” program from the MethPipe package v.3.4.2 (Fang et al. 2012). This program scans the genome using a sliding window approach and fits two models to each interval, one model predicts the methylation levels of each window are the same for both alleles and a second model predicts the methylation levels are different for each allele. The likelihood of the two models is then compared and a false discovery rate corrected *P* value is generated per window (Fang et al. 2012). Sample input files were merged by reproductive group in order to increase the coverage per CpG as this method does not take replication into account. Windows were defined as three CpGs with a minimum coverage of ten reads per CpG. Only regions within the main 18 linkage groups of the *B. terrestris* genome were tested for allele-specific methylation as the program is not designed to cope with the number of unplaced scaffolds (5,591) that the current genome build contains. Finally, regions with allele-specific methylation falling within a gene were annotated with the gene identifier using a custom R script.

This method of identifying regions with allele-specific methylation is preferable compared with using SNP data to identify alleles for the data presented here. Firstly, it is difficult to call SNPs reliably from bisulfite data, this is because C/T SNPs and C/T conversions introduced during bisulfite treatment appear the same within the data (Liu et al. 2012). Secondly, as the samples used were pooled females, each sample may contain multiple SNPs at a given loci meaning the coverage produced per SNP would be too low to produce any reliable estimates of allelic methylation.

### Gene Ontology Analysis

Gene ontology terms for *B. terrestris* were taken from a custom database made in Bebane et al. (2019). GO enrichment analysis was carried out using the hypergeometric test with Benjamini–Hochberg (Benjamini and Hochberg 1995) multiple-testing correction, *q* < 0.05. GO terms from genes showing allele-specific expression were tested for enrichment against a database made from the GO terms of all genes identified in the RNA-Seq data. GO terms from genes showing allele-specific methylation were tested for enrichment against a database made from the GO terms of all genes identified as methylated. Genes were determined as methylated if they had a mean weighted methylation level (Schultz et al. 2012) greater than the bisulfite conversion error rate of >0.05. Descriptions of GO terms and treemaps were generated by REVIGO (Supek et al. 2011).

### Relationship between Allele-Specific Expression and Allele-Specific Methylation

Significant overlap between genes showing allele-specific expression and allele-specific methylation was tested using a hypergeometric test. Overlap plots were generated using the *UpSetR* package in R (Lex et al. 2016). Custom R scripts were used to test for a relationship between allele-specific expression and genes with allele-specific methylation and the interaction of that relationship with reproductive state.

## Results

### Allele-Specific Expression

All reads had 13-bp trimmed from the start due to base bias generated by the Illumina protocol (Krueger et al. 2011). The mean number of uniquely mapped reads was 89.4% ± 0.8% (mean ± SD). This equated to a mean of 10,115,366 ± 1,849,600 uniquely mapped reads (supplementary 1.0.0, Supplementary Material online). The average number of heterozygous SNPs called per sample was 17,753 ± 6,840, of which an average of 9,355 ± 3,781 had a coverage >10 and after filtering to remove potentially homozygous SNPs the average final number of SNPs per sample was 9,297 ± 3,755 (supplementary 2.0, fig. S2*a*, Supplementary Material online). The average number of genes with at least one SNP per sample was 2,436 ± 947 (supplementary 2.0, fig. S2*b*, Supplementary Material online).

Only genes present in at least two colonies per reproductive status were tested for allele-specific expression, this lead to a final conservative list of 2,673 genes (24.2% of all annotated genes in the reference genome Bter_1.0). A total of 139 genes were found to show significant allele-specific expression bias (*q* < 0.05 and average allelic expression proportion >0.65) (supplementary 1.0.1 and 2.0, fig. S3, Supplementary Material online). As expected there were many genes which show a significant *q* value below the cut-off threshold of 0.65 (supplementary 2.0, fig. S4, Supplementary Material online).

**Fig. 2.**
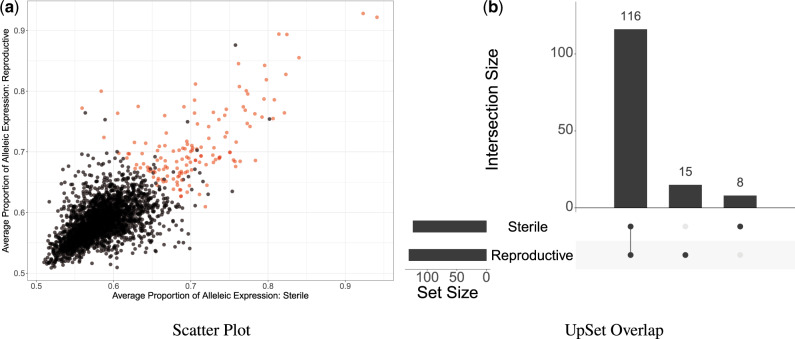
(*a*) Scatter plot showing the allele-specific expression proportion of sterile workers plotted against the allele-specific expression proportion of reproductive workers (the allele-specific expression proportion was averaged across colonies). Each point is a gene, the red points indicate genes showing significant allele-specific expression (*q*<0.05 and average allele-specific expression proportion >0.65). (*b*) An UpSet plot showing the number of genes with allele-specific expression shared by worker reproductive state and the number unique to reproductive or sterile workers (intersection size), indicated by a joint dot or single dot, respectively. The set size shows the total genes with allele-specific expression in either reproductive or sterile workers.

**Fig. 3.**
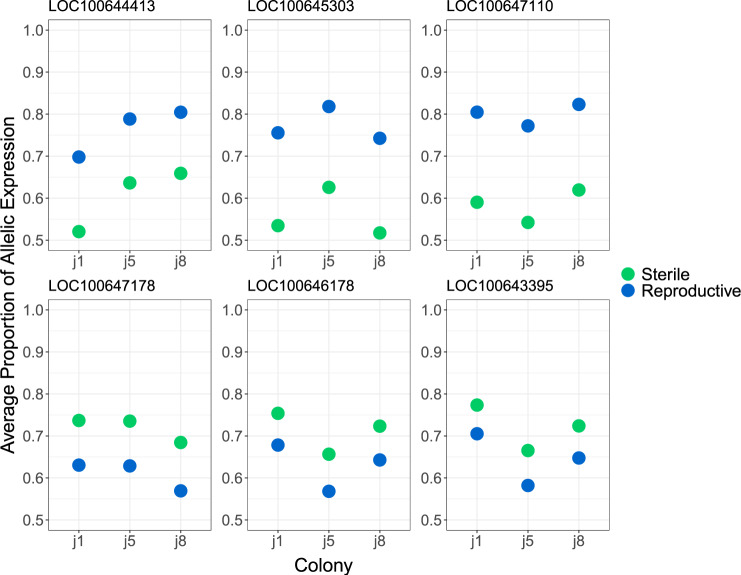
The average proportion of allele-specific expression for genes found to show significant allele-specific expression in only sterile or reproductive workers across colonies. The top row shows the genes with the highest allele-specific expression bias in reproductive workers compared with sterile workers. The bottom row shows the highest allele-specific expression bias in sterile workers compared with reproductive workers.

The genes of reproductive and sterile workers show similar levels of allelic expression (Spearman’s rank correlation, S = 1229363078, rho = 0.61, *P* < 0.0001, fig. 2*a*). Of the 139 genes found to show allele-specific expression a significant number are shared between reproductive and sterile workers (hypergeometric test *P* < 0.0001, fig. 2*b*), with eight found only in sterile workers and 15 found only in reproductive workers (e.g., fig. 3 and supplementary 1.0.1, Supplementary Material online).

**Fig. 4.**
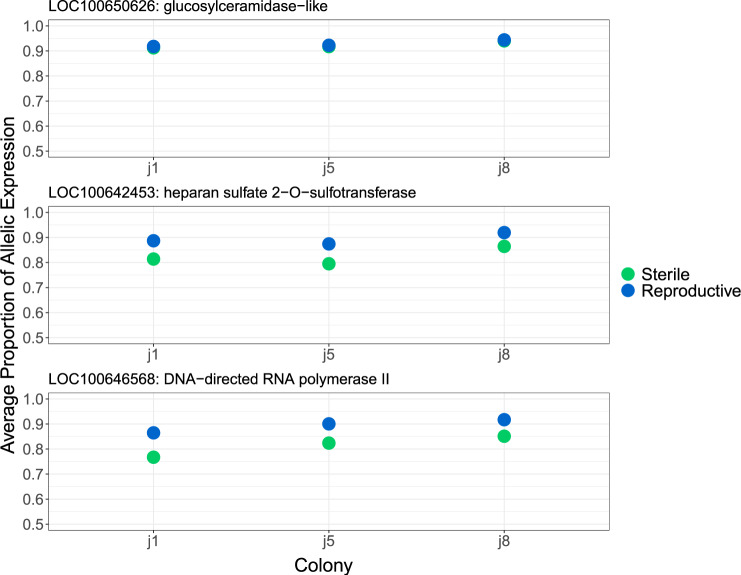
The average proportion of allelic expression for genes found to show the most extreme allele-specific expression in both sterile and reproductive workers across colonies.

There is also some variability in allelic expression proportion between colonies, with reproductive and sterile workers showing similar levels of bias compared with other colony replicates (supplementary 2.0, fig. S5, Supplementary Material online and fig. 3). However, this is less apparent in the most highly biased genes (fig. 4).

Enriched GO terms associated with genes showing significant allele-specific expression were highly varied in both reproductive and sterile workers and were involved in multiple biological processes, some relevant terms include; “female gamete generation” (GO: 0007292), “positive regulation of ovulation” (GO: 0060279) and “histone H3-K27 acetylation” (GO: 0043974) (see supplementary 1.0.2, Supplementary Material online, for all enriched terms).

GO terms enriched for the eight genes showing allele-specific expression in sterile workers included mostly catabolic processes, but also “response to pheromone” (GO: 0019236). The GO terms enriched for the 15 genes showing allele-specific expression in reproductive workers included; “primary sex determination” (GO: 0007538) as well as multiple other cellular processes, supplementary 1.0.2, Supplementary Material online. These results should be interpreted with care as the gene lists are relatively small. However, it is worth noting that the hypergeometric test used to generate the enriched terms has been previously shown to be the most appropriate statistic for gene ontology enrichment for small gene lists (Rivals et al. 2007).

### Allele-Specific Methylation

Up to a maximum of 10 bp were trimmed from the start of all reads due to base bias generated by the Illumina sequencing protocol (Krueger et al. 2011). The mean mapping efficiency was 63.6% ± 1.4% (mean ± SD) and the mean coverage was 17.7 ± 0.5 reads per base, the average number of uniquely mapped reads were 27,709,214 ± 753,203 (supplementary 1.0.3, Supplementary Material online). 12.79% of the genome was not tested for allele-specific methylation as only regions in the main 18 linkage groups of the *B. terrestris* genome (Bter_1.0) could be tested.

Reproductive workers have significantly more regions with allele-specific methylation compared with sterile workers, 303 (supplementary 1.0.4, Supplementary Material online) compared with 201 (supplementary 1.0.5, Supplementary Material online), respectively (χ^2^ goodness of fit; χ^2^= 20.643, df = 1, *P* < 0.0001). The majority of these regions occur within annotated genes, 26 and 15 regions with allele-specific methylation occur outside of a gene for reproductive and sterile workers. Additionally, a small number of regions overlap multiple gene annotations, ten in reproductive workers and nine in sterile workers.

**Fig. 5.**
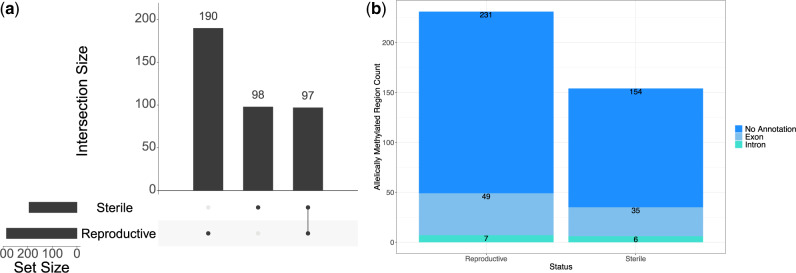
(*a*) UpSet plot showing the number of genes with allele-specific methylation in just reproductive and sterile workers, as well as the number of genes in common between both reproductive states. (*b*) Component bar plot showing the number of regions with allele-specific methylation within genes, found in exons and introns and the number without additional annotation.

Most genes with allele-specific methylation are unique to either sterile or reproductive workers, however, there is a significant number of common genes with allele-specific methylation (hypergeometric test *P* < 0.0001, fig. 5*a*). Most regions with allele-specific methylation found within genes do not have additional annotation, however, there are more located in exons compared with introns for both reproductive and sterile workers (fig. 5*b*).

Enriched GO terms associated with genes with allele-specific methylation in both reproductive states are involved in a large variety of biological processes with many relating to the term “positive regulation of RNA splicing” (GO: 0033120). As above, the enriched GO terms associated with genes with allele-specific methylation in just sterile or reproductive workers are also involved in a large number of biological processes. However, the terms “oocyte development” (GO: 0048599), “ovarian follicle development” (GO: 0001541), “oogenesis stage” (GO: 0022605), and other reproductive terms were enriched in genes with allele-specific methylation of reproductive workers. Additionally, none of these terms was identified in the GO terms associated with the genes with allele-specific methylation in sterile workers (supplementary 1.0.6, Supplementary Material online). Other reproductive-related GO terms were associated with genes with allele-specific methylation in sterile workers but not reproductive workers, such as “ovarian nurse cell to oocyte transport” (GO: 0007300) and “germ cell development” (GO: 0007281).

### Relationship of Allele-Specific Expression and Methylation

**Fig. 6.**
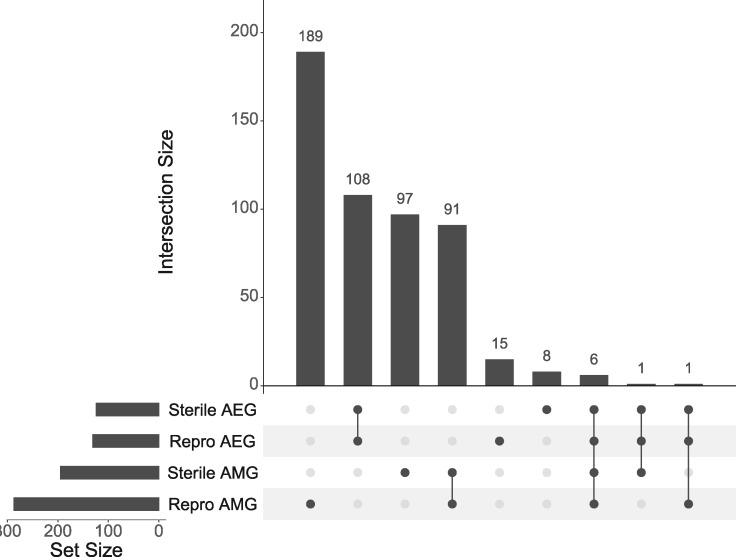
(*a*) UpSet plot showing the overlapping genes identified as having allele-specific methylation and/or allele-specific expression in both reproductive and sterile workers. AEG, allelically expressed gene; AMG, allelically methylated gene.

There is no significant overlap between genes showing allele-specific expression and allele-specific methylation (overlap between all conditions; hypergeometric test *P* = 0.209, fig. 6). However, six genes were found to show allele-specific methylation and expression in both reproductive states, one gene was found to show allele-specific expression in both states and allele-specific methylation in reproductive workers and one gene shows allele-specific expression in both states and allele-specific methylation in sterile workers (table 1).

**Table 1:** Genes Identified as Showing Allele-Specific Methylation and Expression in Both Reproductive and Sterile Workers

Gene ID	Gene Description
LOC100643777	40S ribosomal protein S6
LOC100643941	Connectin
LOC100644811	Neuroligin-4, Y-linked
LOC100652132	Importin-11
LOC100644932	AP-1 complex subunit mu-1
LOC105665778	Regulator of microtubule dynamics protein 1-like
LOC105666711^a^	Tyrosine-protein kinase Btk29A^a^
LOC100643219^b^	Putative pre-mRNA-splicing factor ATP-dependent RNA helicase PRP^b^

a This gene does not show allele-specific methylation in reproductive workers.

b This gene does not show allele-specific methylation in sterile workers.

The GO terms enriched for the genes found with allele-specific methylation and expression (table 1) compared with the entire genome as background, included a large variety of biological processes (supplementary 1.0.7, Supplementary Material online). Specifically, some reproductive-related terms were also enriched; “female germline ring canal formation” (GO: 0007301) and “ovarian fusome organization” (GO: 0030723).

**Fig. 7.**
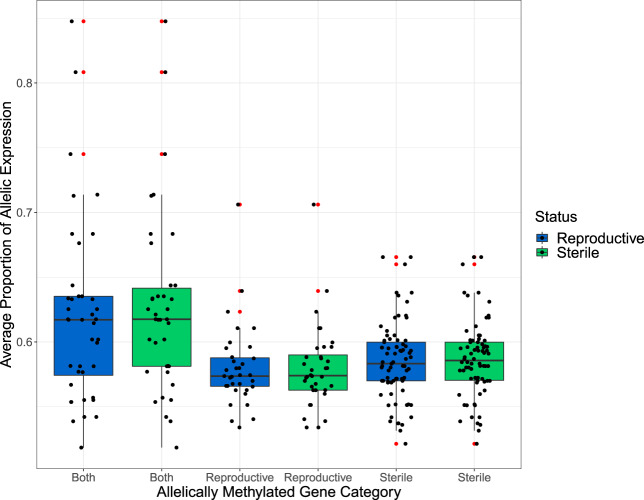
Boxplots showing the proportion of allele-specific expression in reproductive and sterile workers for genes identified with allele-specific methylation in: both reproductive states, just reproductive workers or just sterile workers. Each boxplot shows the median along with the 25th and 75th percentiles. The whiskers represent 1.5× the interquartile range. Outliers are represented as additional red points and each gene is represented by a black dot.

There is a significant difference in the proportion of allele-specific expression of genes with allele-specific methylation in either reproductive workers, sterile workers, or both (Kruskal–Wallis; χ^2^ = 28.838, df = 2, *P* < 0.0001). Genes with allele-specific methylation in both reproductive and sterile workers show on an average higher levels of allele-specific expression compared with those unique to either reproductive or sterile workers (Dunn test with Benjamin–Hochberg correction; both compared with unique in reproductive workers *Z* = 5.149, *q* < 0.0001, both compared with unique in sterile workers *Z* = 4.147, *q* < 0.0001) (fig. 7). Additionally, genes with allele-specific methylation unique to reproductive workers show similar levels of allelic expression compared with genes with allele-specific methylation unique to sterile workers (Dunn test with Benjamin–Hochberg correction; reproductive compared with sterile *Z* = −1.851, *q* = 0.06) (fig. 7). Finally, there is no interaction between reproductive state and allele-specific expression proportion on the allele-specific methylation status of a gene (ANOVA, interaction vs. main effects model, *F*_2,296_ = 0.1094, *P* = 0.896) (fig. 7).

## Discussion

Using whole-genome bisulfite sequencing and RNA-seq from reproductive and sterile *B. terrestris* workers from three independent colonies, we have identified genome-wide allele-specific expression and allele-specific methylation. We found no significant overlap between genes which display both allele-specific methylation and allele-specific expression indicating *cis*-acting DNA methylation does not directly influence allele-specific expression in this species. We also found the majority of genes displaying allele-specific expression are common between reproductive and sterile workers and the proportion of allele-specific expression generally varies between colonies. This suggests allele-specific expression is relatively stable between worker phenotypes and that the underlying genotype may influence allele-specific expression in some cases. Additionally, allele-specific methylation appears less stable between worker phenotypes, with reproductive workers showing significantly more genes with allele-specific methylation. Finally, we have found that genes with common allele-specific methylation between reproductive and sterile workers show a higher proportion of allele-specific expression bias compared with genes with allele-specific methylation unique to either just reproductive or sterile workers. This is suggestive of multiple roles of allele-specific methylation in this species.

This study has identified 139 genes which show allele-specific expression from a stringent subset of genes covering 24% of all annotated genes within the *B. terrestris* genome. This number is in line with previous research that identified ∼500 loci across the whole genome of *B. terrestris* (Lonsdale et al. 2017). The proportion of allelic expression bias differed between colonies and the GO terms enriched for all genes with allele-specific expression were varied. This indicates allele-specific expression plays a diverse role in *B. terrestris* and some instances may be a product of the underlying genotype. Previous research identified 61 genes showing allele-specific expression in a cross of two *Nasonia* species, the expression bias in all genes was attributed to *cis*-effects (Wang et al. 2016). Given that each colony used here is genetically distinct, *cis*-effects, such as SNPs, are likely represented in the results.

Although the majority of genes showing allele-specific expression were common between reproductive and sterile workers, a large number of genes show allele-specific methylation which is unique to either reproductive or sterile workers. Additionally, there are significantly more sites with allele-specific methylation in reproductive workers compared with sterile workers, with genes with allele-specific methylation in both sterile and reproductive workers enriched for different GO terms related to reproduction. These findings support previous research which suggests methylation is associated with worker reproductive behavior. Amarasinghe et al. (2014) found a global erasure of DNA methylation increased reproductive behavior, Liu et al. (2018) found differences in expression in genes responsible for methylation between castes and Marshall et al. (2019) found differentially methylated genes between *B. terrestris* reproductive and sterile workers, some of which were involved in reproductive processes. Numerous other studies have linked methylation to caste differences in various other social insect species, such as; *Apis mellifera* (Elango et al. 2009; Lyko et al. 2010), *C. floridanus* and *H. saltator* (Bonasio et al. 2012), *Polistes dominula* (Weiner et al. 2013), and *Zootermopsis nevadensis* (Glastad et al. 2016). However, the statistical validity of many of these studies has been questioned (Libbrecht et al. 2016). The development of experimental techniques to alter DNA methylation, such as CRISPR/Cas (Vojta et al. 2016), will allow for experiments to test the causal effect of DNA methylation and allele-specific methylation on caste determination in social insects.

It is, however, clear from this study that DNA methylation does not play a direct causal role in the production of all allele-specific expression events, with only a small number of genes displaying both allele-specific expression and methylation. Lonsdale et al. (2017) found 19 genes which displayed both allele-specific methylation and expression, none of which corresponds to the genes identified here. This may be because different tissue types were used, Lonsdale et al. (2017) used whole body whereas here we used head tissue. Allele-specific expression and methylation have been shown to vary dramatically by tissue type and developmental stage (Babak et al. 2015) which may explain some of the lack of agreement between studies. Additionally, Lonsdale et al. (2017) used only a single individual and as discussed above allele-specific expression and methylation can be caused by the underlying genotype. Finally, we utilized whole-genome bisulfite sequencing whereas Lonsdale et al. (2017) enriched their sequencing libraries using antibodies specific to methylated and unmethylated cytosines, it is therefore likely the greater resolution of bisulfite sequencing has allowed us to identify more sites with allele-specific methylation.

There has been a recent focus on identifying imprinted genes in Hymenopteran species as an independent test for Haig’s kinship theory (Pegoraro et al. 2017). Parent-of-origin allele-specific expression has been identified in both honeybees (Kocher et al. 2015; Galbraith et al. 2016; Smith et al. 2020) and bumblebees (Marshall et al. 2020) and there has been speculation that DNA methylation may act as an imprinting mark. The results of this study do not support this idea due to the lack of association between allele-specific DNA methylation and allele-specific expression. However, this does not completely rule out the possibility that methylation may act as an imprinting mark if only a small number of genes are actually imprinted, as in humans (Tycko 2010). Although, this requires further investigation utilizing reciprocal crosses to identify parent-of-origin DNA methylation. Additional imprinting marks should also not be ruled out as GO terms enriched for genes showing allele-specific methylation here included histone modifications. Genes displaying allele-specific methylation may feed into other mechanisms which may, in-turn, drive allele-specific expression, accounting for the lack of direct association. For example, methylation of an imprinting control region can signal certain histone modifications which can allow the formation of condensed chromatin, silencing many genes in one region (Barlow 2011), this process can also occur in an allele-specific manner (Tycko 2010).

Although only a small number of genes show allele-specific methylation and allele-specific expression, genes showing allele-specific methylation in both reproductive and sterile workers had higher allelic expression bias compared with those found only in one worker type. One explanation is that genes with allele-specific methylation present in both reproductive and sterile workers carry out different functions to those identified in a single worker type. This is supported by the diverse GO terms obtained for shared and reproductive/sterile-specific genes with allele-specific methylation. In humans, the majority of allele-specific methylation is genotype dependent rather than parentally inherited (Meaburn et al. 2010). Whereas, allele-specific methylation associated with imprinting may change at different stages of development (Edwards et al. 2017). It may therefore be that the common genes with allele-specific methylation identified here are linked to genotype (i.e., epialleles) whereas the reproductive state-specific genes with allele-specific methylation may represent imprinting marks. However, this is speculation and requires further investigation.

In order to further understand the role and origin of allele-specific methylation a pipeline is needed which integrates SNP data (generated from genomic DNA), to allow the identification of specific alleles. Using this method rather than the probabilistic models employed here would enable hyper/hypomethylation (i.e., higher or lower methylation in one condition compared with another) to be associated with allele-specific expression when they occur in tandem. Additionally, this method, with increased biological replication per colony, would facilitate the identification of epialleles, that is, when allele-specific methylation is driven by genotype. Epialleles have been identified in the honeybee (Wedd et al. 2016; Yagound et al. 2019) and will be important in the identification of parent-of-origin methylation (Remnant et al. 2016).

Overall this study has identified genome-wide allele-specific expression and allele-specific methylation in reproductive and sterile bumblebee workers from three genetically distinct colonies. We have found *cis*-acting allele-specific DNA methylation does not directly influence allele-specific expression. We have also found differences in allele-specific expression between colonies indicating a possible role for the underlying genotype. Finally, we have identified a small number of genes which show allele-specific expression in just reproductive or sterile workers and a much large number which show allele-specific methylation unique to each phenotype. The results of this study have implications for the functional role of DNA methylation in genomic processes such as imprinting, gene expression regulation, and caste determination in social insects. 

## Supplementary Material

evaa132_Supplementary_Data
